# The competence in innovation and entrepreneurship education among Chinese university teachers: a latent profile analysis

**DOI:** 10.3389/fpsyg.2026.1703298

**Published:** 2026-04-23

**Authors:** Wenyi Zhang, Huiqing Li, Xudan Zhang, Zhiying Yang, Yingzhi He

**Affiliations:** 1 School of Business Administration/Cantonese Merchant School/Innovation and Entrepreneurship School, Guangdong University of Finance and Economics, Guangzhou, China; 2 The Chinese University of Hong Kong, Shenzhen, Shenzhen, China; 3 School of Education, South China Normal University, Guangzhou, China

**Keywords:** higher education, innovation and entrepreneurship education, latent profile analysis, teacher competence, university teacher

## Abstract

With the rapid expansion of innovation and entrepreneurship education (IEE), understanding the competencies of university teachers in this field has become increasingly important. This study employs the latent profile analysis (LPA) to identify and examine distinct competency subgroups among university IEE teachers. The study surveyed 396 university teachers specializing in IEE in China. The findings indicated that IEE competencies encompassed eight dimensions: entrepreneurial mindset, professional mindset, ambition, optimism, self-efficacy, professional knowledge, enterprising skills, and teaching ability. Latent profile analysis identified three distinct subgroups of IEE teachers: the high IEE-competent group, the average IEE-competent group, and the moderate IEE-competent group. Moreover, multinomial logistic regression analysis demonstrated that university IEE teachers with prior experience in entrepreneurship management or innovation-entrepreneurship research possessed significantly stronger instructional competencies in IEE than those without such knowledge. These results provide empirical evidence linking teachers' personal and professional backgrounds to their IEE instructional practices, and highlight the critical role of experiential learning in developing pedagogical competence.

## 1 Introduction

Innovation and entrepreneurship education (IEE) represents a distinct educational paradigm that integrates entrepreneurial theory with the development of practical skills. It encompasses two complementary dimensions: conceptual innovation and practical implementation (Gao and Cao, 2007; Ma et al., 2020). Innovation provides the conceptual foundation for entrepreneurial activities, while entrepreneurship serves as the mechanism through which innovative ideas are realized. The development of IEE has varied across regions. The United States was a pioneer in IEE, and the United Kingdom has made rapid progress. In Asia, Japan represents a relatively mature system of IEE, while China, despite its late inception, has experienced rapid development in recent years (Ma et al., 2020). In the contemporary context, sustainable development has emerged as a core imperative for the 21st century, shaping both entrepreneurial ventures and IEE programs (Shu et al., 2020). Driven by technological progress and globalization, governments and educational institutions worldwide have prioritized IEE as a strategic driver of regional economic development and societal welfare (Wang and Fu, 2023).

Research on the implementation of IEE in higher education institutions has expanded in both volume and scope globally. Internationally, scholars emphasize that the principles of IEE apply not only to students but also to educational institutions, requiring both institutional leadership and faculty to embrace an entrepreneurial mindset (Harkema and Schout, 2008). Furthermore, the shift to effective IEE relies heavily on the adoption of innovative instructional methodologies that transcend traditional disciplinary boundaries (Samuel and Rahman, 2018). In the broader international context, educators are increasingly viewed not merely as knowledge disseminators, but as entrepreneurial role models whose personal engagement directly shapes students' entrepreneurial intentions (San-Martín et al., 2021). Aligned with these global trends, government initiatives and university reforms in China have provided strong support for IEE, underscoring the need to cultivate qualified IEE practitioners across all academic levels (Ministry of Education of the People's Republic of China, 2024). Regardless of the regional context, effective educational innovation requires considering teacher-related instructional factors (Zhao and Zhang, 2021). Bottom-up policy design facilitates large-scale technological integration by engaging key stakeholders—particularly teachers and administrative staff—as critical change agents within the IEE ecosystem (De Voto and Thomas, 2020; San-Martín et al., 2021).

### 1.1 Teacher competencies and the iceberg model in IEE

In China, there has been a growing emphasis on the pedagogical competence of higher education teachers in IEE, including the factors that shape instructional competency (Liu et al., 2024). Competence is broadly defined as the integrated knowledge, skills, and attributes required for effective job performance (Gupta, 1999; Katane et al., 2006). In the teaching context, Vogt and Rogalla (2009) identify four critical instructional competencies such as subject knowledge, diagnostic ability, and teaching methods. Besides, research on higher education teacher competencies typically clusters around six key dimensions like personality, professionalism, pedagogy, and research capability (Dervenis et al., 2022). However, existing research has devoted limited attention to examining the multifaceted IEE competence of higher education teachers. To address this gap, this study conceptualizes IEE teacher competency using the Competency Iceberg Model (Spencer and Spencer, 2008). This theory metaphorically conceptualizes human competence as an iceberg, positing that individual characteristics fall into two distinct categories based on their visibility and malleability. The model partitions human competence into two tiers: surface-level observable attributes (knowledge and skills) and deep-level unobservable psychological characteristics (motives, traits, and self-concepts). Its core premise is that while observable skills form the foundational layer, these underlying unobservable traits ultimately drive superior performance in highly complex and uncertain professional contexts.

Guided by this theoretical framework, we identified eight specific competency dimensions for IEE teachers and explicitly aligned them with the structural components of the Iceberg Model. The observable competencies encompass professional knowledge (knowledge), enterprising skills, and teaching ability (skills)—all of which constitute foundational pedagogical expertise. Critically, the unobservable competencies comprise ambition (motives), optimism (traits), and three self-concept dimensions: entrepreneurial mindset, professional mindset, and self-efficacy. By clearly articulating this observable-unobservable alignment, our conceptual framework advances upon prior models that focus primarily on observable instructional skills. This represents a novel conceptual contribution by demonstrating how deep-seated intrinsic psychological traits (unobservable) serve as the core theoretical drivers of effective instructional behaviors (observable) in IEE practice.

University teacher competency in IEE is influenced by multiple factors. Liu et al. (2024) suggest teacher professional development can be demonstrated through cognitive enhancement and subsequent behavioral implementation. Externally, improvements in training environments and investments in personal entrepreneurial behaviors—such as innovation, autonomy, and resource management—are particularly vital for enhancing teacher entrepreneurship competency (Zhu et al., 2023). Accordingly, teaching tenure, entrepreneurship/business management experience, IEE research experience, and competition mentoring experience may be significantly associated with the IEE competence of higher education teachers.

### 1.2 A person-centered approach: latent profile analysis

Despite these theoretical advances, extant empirical research assessing IEE competence has primarily employed variable-centered approaches, which often overlook significant individual heterogeneity among IEE educators. To address this limitation, we adopted Latent Profile Analysis (LPA), a person-centered statistical technique that identifies distinct subgroups within a sample based on observed continuous variables (Spurk et al., 2020). As a person-centered approach, LPA has been effectively applied to study motivation and emotional labor at the individual level, providing empirical evidence for targeted interventions (Yin et al., 2020; Zhao et al., 2023).

### 1.3 The current study

Overall, the present study addresses two core research objectives: first, to explore the latent competence profiles of higher education IEE teachers based on the eight-dimensional competency framework; and second, to systematically examine potential factors associated with specific competence profiles, including teaching tenure, entrepreneurship/business management experience, IEE research experience, and competition mentoring experience. While prior research has examined teacher competency models and their associated influencing factors (Liu et al., 2024), the application of LPA is particularly warranted given the significant individual differences among IEE teachers. Identifying and examining these teacher subgroups can more effectively facilitate personalized professional development for IEE teachers and ultimately enhance the overall quality of IEE in higher education institutions.

## 2 Materials and methods

### 2.1 Participants and procedure

The study received ethical approval from the HSJKY Scientific Research Ethics Committee (approval number: HSJKY-EDU-2022-001). Participants were recruited from five distinct groups: (a) full-time higher education educators who teach IEE courses; (b) faculty members who mentor students in entrepreneurship competitions and entrepreneurial projects; (c) university administrators responsible for overseeing student entrepreneurship programs; (d) student affairs personnel (e.g., academic advisors and staff from student employment and entrepreneurship service centers); and (e) researchers specializing in IEE scholarship. Within China's “Whole-person Education” (Sanquan Yuren) system, student affairs personnel hold a dual role as both educators and administrators (Ministry of Education of the People's Republic of China, 2017). Given the applied nature of IEE, these administrative staff and researchers often serve as primary mentors for entrepreneurial projects (Zhu and Shu, 2021; Zhu et al., 2023). Classifying these individuals as “IEE educators” aligns closely with international perspectives, which emphasize that effective IEE requires a holistic institutional approach that actively engages institutional leadership and diverse practitioners (Harkema and Schout, 2008; San-Martín et al., 2021). The study targeted these participants and employed an online questionnaire survey. The online questionnaire was designed and distributed through the Chinese professional online research platform *Wenjuanxing* (www.wjx.com). The questionnaire link was distributed within IEE educator communities via both individual outreach and institutional dissemination.

Participants were recruited from across mainland China, with a primary focus on Guangdong Province. Data collection took place between February and March 2023, yielding a total of 396 valid responses. Participants ranged in age from under 30 to over 60 years, with the sample comprising 138 males (34.85%) and 258 females (65.15%). IEE engagement tenure among participants was distributed as follows: the largest proportion (45.7%, *n* = 181) reported less than 1 year of IEE involvement, followed by 38.6% (*n* = 153) with 1–5 years of experience. A smaller proportion of participants (8.6%, *n* = 34) had 6–10 years of IEE engagement, while equal proportions (3.5% each, n = 14 per group) reported 11–15 years and more than 15 years of IEE tenure, respectively. With respect to entrepreneurship/business management experience, 35.9% (*n* = 142) of participants reported having such experience, while 64.1% (*n* = 254) did not. Similarly, 51.5% (*n* = 204) of participants reported prior involvement in leading or participating in IEE-related research projects, while 48.5% (*n* = 192) reported no such experience. For competition mentoring experience, 45.2% (*n* = 179) of participants reported mentoring students in IEE competitions, while 54.8% (*n* = 217) reported no such experience. All participants participated voluntarily and provided written informed consent.

### 2.2 Measures

The Innovation and Entrepreneurship Education Competence Questionnaire (IEEC) was developed for this study via a rigorous process that integrated competency theory, in-depth semi-structured interviews, and expert review by senior IEE educators (full- and part-time) and university psychology faculty with expertise in competency research.

Firstly, the IEEC was developed to assess IEE competence among higher education IEE educators. The initial IEEC comprised 203 items across four core dimensions: (1) personality traits (141 items); (2) IEE professional knowledge (16 items); (3) enterprising skills (21 items), which assess practical innovation competence; and (4) teaching ability (25 items), which measures instructional skills. All items were rated on a 5-point Likert scale, with response options ranging from 1 (Strongly Disagree) to 5 (Strongly Agree). To mitigate social desirability bias, items 9, 10, 15, 19, 24, 37, 42, 45, 54, 59, 63, 65, 85, 89, 93–95, 99–100, 118, 122–123, 127, and 130–131 were reverse-coded.

Secondly, the initial IEEC was administered via an online survey platform to IEE educator communities to conduct a pilot study. A total of 358 surveys were collected during the pilot phase, of which 350 were valid, yielding an effective response rate of 97.78%. The pilot sample comprised 31.14% males and 68.86% females. Most pilot participants were aged 30–50 years, and the majority were affiliated with higher education institutions in the Guangdong-Hong Kong-Macao Greater Bay Area (GBA). To evaluate the IEEC's psychometric properties, pilot participants were split into high- and low-score groups based on total scale scores, and item score differences between these groups were examined. To evaluate the IEEC's psychometric properties, pilot participants were split into high- and low-score groups based on total scale scores, and item score differences between these groups were examined. *Items with non-significant group differences were deemed to have low discriminant validity for the target construct; conversely, items with significant group differences demonstrated strong discriminant powe*r. If no significant difference was observed, the item was considered to have weak discrimination for the underlying trait; conversely, a significant difference indicated strong discriminatory power. Next, the correlation between each item score and the total score (item–total correlation) was used as an indicator of item discrimination. Items with higher item-total correlations demonstrated greater ability to differentiate the target construct, and vice versa. Items with higher correlations with the total score were considered to have greater ability to distinguish the underlying trait, and vice versa. Finally, exploratory factor analysis (EFA) was conducted, and items with factor loadings below a predetermined threshold were removed to refine the scale. Ultimately, 116 items were removed, resulting in a refined 87-item IEEC that aligned with the study's research objectives.

Thirdly, the refined 87-item IEEC assesses four key competence dimensions, with subdimensions and item counts as follows: (1) Personality Traits (5 subdimensions: 16-item Entrepreneurial Mindset, 4-item Professional Mindset, 4-item Ambition, 3-item Optimism, and 6-item Self-Efficacy [adapted from the General Self-Efficacy Scale]); (2) IEE Professional Knowledge (12 items); (3) Enterprising Skills (18 items), which assess practical innovation competence; and (4) Teaching Ability (24 items), which measures instructional skills. All items employ a 5-point Likert scale (1 = strongly disagree to 5 = strongly agree) with total scores calculated by summation. The IEEC demonstrated excellent internal consistency reliability, with Cronbach's α coefficients exceeding 0.90 across all dimensions (Personality Traits: 0.94; IEE Professional Knowledge: 0.92; Enterprising Skills: 0.95; Teaching Ability: 0.96) and an overall scale α of 0.98. Confirmatory factor analysis (CFA) confirmed acceptable model fit for the IEEC, with the following fit indices: χ^2^/ df = 2.921, AIC = 75,843.177, BIC = 76,926.808, aBIC = 76,063.744, SRMR = 0.073, and RMSEA = 0.069 (90% CI [0.067, 0.070]). These indices confirm acceptable model fit for the measure.

### 2.3 Data analyses

In this study, latent profile analysis was conducted using Mplus 8.3 software. The data analysis was carried out in three steps. Given the IEEC's eight dimensions vary in item count, a weighted scoring approach and linear score transformation were applied to ensure score comparability across dimensions. Therefore, this study adopted a weighted scoring approach and applied a linear transformation to the scores to enhance comparability across dimensions. Specifically, the raw total score for each dimension was multiplied by 0.125, divided by the theoretical maximum score of the respective dimension, and subsequently multiplied by 100. The coefficient of 0.125 was derived from the IEEC's eight dimensions, with each dimension accounting for one-eighth of the total composite score.

In the first step, the demographic survey items were recoded as follows: (1) The item “years of IEE teaching experience” was dichotomized: “less than 1 year” and “1–5 years” were recoded as 0 (indicating 5 years or less of teaching experience), and “6–10 years”, “11–15 years”, and “more than 15 years” were recoded as 1 (indicating more than 5 years of teaching experience), due to the relatively small sample size in these latter categories; (2) The item “whether has the entrepreneurial or business management experience” was recoded as 0 for ‘No” and 1 for “Yes”; (3) The item “whether has experience in conducting research on innovation and entrepreneurship education” was similarly recoded as 0 for “No” and 1 for “Yes”; (4) The item “whether has experience in guiding students to participate in innovation and entrepreneurship competitions” was also recoded as 0 for “No” and 1 for “Yes”.

In the second step, a latent profile model was estimated to identify IEE competence subgroups among higher education IEE teachers. Latent Profile Analysis is a person-centered analytical approach that uses latent categorical variables to account for relationships among observed continuous indicators. The underlying assumption of LPA is that the probability distribution of responses to observed variables can be explained by a small number of mutually exclusive latent categories. In the current study, the dimensions of IEE competencies included “entrepreneurial mindset, professional mindset, ambition, optimism, self-efficacy, professional knowledge, enterprising skills, and teaching ability”. This step began with an initial model and progressively increased the number of categories in the latent profile model until the model that the best-fitting model was identified.

In the third step, building on the results from the second step, latent profile class membership was treated as the dependent variable. The independent variables included teaching experience, entrepreneurial or business management experience, experience in innovation and entrepreneurship research, and experience in guiding students in innovation and entrepreneurship competitions. A multinomial logistic regression model was estimated to examine the predictive effects of these four independent variables on latent profile class membership of teachers' IEE competence.

Model fit for LPA was evaluated using the following criteria: (1) Akaike Information Criterion (AIC), Bayesian Information Criterion (BIC), and adjusted Bayesian Information Criterion (aBIC), where lower values indicate superior model fit; (2) Entropy, which ranges from 0 to 1, with values closer to 1 indicating more accurate latent class classification. An Entropy value of 0.6 indicates a classification accuracy of over 80%, and a value of 0.8 indicates an accuracy of over 90% (Nylund et al., 2007); (3) The Lo-Mendell-Rubin likelihood ratio test (LMR) and the bootstrapped likelihood ratio test (BLRT). When the *p*-value is significant, it indicates that the model with *k* categories is significantly better than the model with *k*-1 categories (Scotto Rosato and Baer, 2012). This study comprehensively considered the results of these indices, alongside the practical context of university teachers' IEE competencies to determine the optimal model and to label the categories accordingly. All statistical tests were conducted using two-tailed tests, with a significance level of α = 0.05.

## 3 Results

### 3.1 Exploratory LPA

To explore the latent patterns of university entrepreneurship teachers' IEE competencies, we established latent profile models based on participants' responses across eight dimensions in the questionnaire: entrepreneurial mindset, professional mindset, ambition, optimism, self-efficacy, professional knowledge, enterprising skills, and teaching ability. Beginning with an initial 2-class model, we progressively increased the number of classes and ultimately fitted four latent profile models. The model fit indices are presented in Table 1.

**Table 1:** The goodness-of-fit indexes of exploratory LPA.

Model	AIC	BIC	aBIC	LMR	BLRT	Entropy	Latent class probability
2	11,081.68	11,181.22	11,101.90	^***^	^***^	0.88	0.42/0.58
**3**	**10,692.88**	**10,828.25**	**10,720.37**	**< 0.05**	^***^	**0.91**	**0.13/0.56/0.32**
4	10,551.93	10,723.13	10,586.69	0.50	^***^	0.85	0.24/0.08/0.40/0.28
5	10,431.82	10,638.85	10,473.85	< 0.05	^***^	0.88	0.05/0.22/0.28/0.37/0.09

^***^p < 0.001. Bold type denotes the suggested best model by the respective index.

Table 1 reports the LPA fit indices, which show that AIC, BIC, and aBIC values were the highest for the 2-class model and decreased incrementally as the number of latent classes increased. The most substantial reduction (>3) in AIC, BIC, and aBIC occurred when transitioning to the 3-class model. In the 3-class model, both the LMR and BLRT values were statistically significant, and the Entropy value peaked above 0.90, indicating excellent classification accuracy. The 4-class model showed non-significant LMR values, suggesting the superiority of the 3-class solution. Furthermore, the 5-class model yielded two classes with sample proportions below 10%. Based on both statistical evidence and model parsimony, the 3-class classification for university teachers' IEE competencies profiles was ultimately selected.

**Figure 1:**
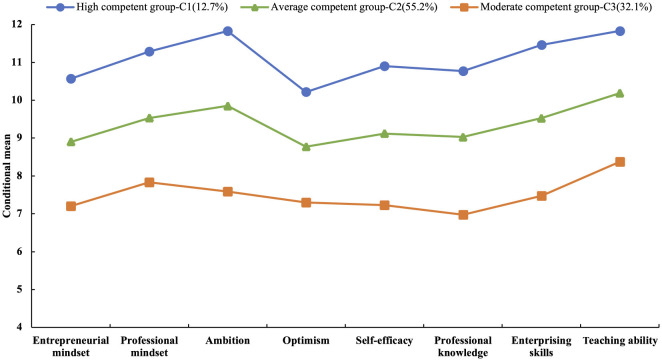
Conditional mean distribution diagram of each latent class.

Conditional mean scores for each of the eight IEEC dimensions across the three latent classes were further examined, and the distribution is presented in Figure 1. As shown in Figure 1, the three latent classes (C1, C2, and C3) exhibited distinct levels of competence across all eight IEEC dimensions. Class 1 (12.7%), labeled the High IEE-Competent Group, exhibited consistently high conditional mean scores across all dimensions, with most values above 10. Class 2 (55.2%), labeled the Average IEE-Competent Group, had conditional mean scores ranging from 8 to 10 across all dimensions. Class 3 (32.1%), labeled the Moderate IEE-Competent Group, had conditional mean scores between 6 and 8 across all dimensions. Accordingly, the three latent classes were formally designated as follows: C1 = High IEE-Competent Group; C2 = Average IEE-Competent Group; C3 = Moderate IEE-Competent Group. The estimated latent class proportions for Classes 1, 2, and 3 were 13%, 56%, and 32%, respectively.

### 3.2 The Influence of Different Experiences on Classification

This study further examined the effects of four categorical independent variables (i.e., teaching duration, entrepreneurship/business management experience, innovation-entrepreneurship research experience, and competition mentoring experience) on the three-category classification of university teachers' IEE competencies using multinomial logistic regression analysis. The “Moderate IEE-competent group” (Category 3) was designed as the reference category to calculate odds ratios (OR) coefficients and quantify the effects of these predictors across the latent profiles. All independent variables were dummy-coded, with teaching duration in IEE using “5 years or less” as the baseline (creating two dummy variables). The other three experience-based variables (i.e., entrepreneurship/business management experience, innovation-entrepreneurship research experience, and competition mentoring experience) used “no experience” as the reference level (each generating one dummy variable), ensuring proper statistical comparison of competency group differences attributable to these professional background characteristics.

The results of the multinomial logistic regression analysis (Table 2) revealed that teachers with: (1) entrepreneurship/business management experience (*p* < 0.05), (2) innovation-entrepreneurship research experience (*p* < 0.05), or (3) competition mentoring experience (marginally significant, *p* = 0.051), were 4.82, 4.72, and 4.70 times more likely, respectively, to belong to the “High IEE-competent group” (C1) than the “Moderate IEE-competent group” (C3). Significant differences were also found between the “Average competency Group” (C2) and “Moderate IEE-competent group” (C3) regarding innovation and entrepreneurship research experience (*p* < 0.05), with teachers having such research experience being 2.11 times more likely to be classified into C2 than C3. Compared to their counterparts without such experience, university teachers with either entrepreneurship/business management experience or innovation-entrepreneurship research experience demonstrate significantly stronger competencies in delivering IEE.

**Table 2:** Multinomial logistic regression of different experiences on each latent class.

Variables	High competent group C1		Average competent group C2	
	OR	CI (95%)	OR	CI (95%)
Teaching duration more than 5 years	1.45	0.21–2.69	0.77	0.27–1.27
**Entrepreneurship/business management experience**	**4.82** ^*^	**1.22–8.42**	1.98	0.94–3.03
**Innovation and entrepreneurship research experience**	**4.72** ^*^	**1.04–8.39**	**2.11** ^*^	**1.1–3.13**
Competition mentoring experience	4.7 (*p* = 0.051)	0.97–8.42	1.22	0.64–1.8

^*^p < 0.05. Bold values indicate statistically significant results.

## 4 Discussion

The global higher education sector has placed increasing emphasis on innovation and entrepreneurship programs, with universities worldwide actively developing initiatives to advance IEE implementation. The IEE competency of university instructors is a critical determinant of successful innovation and entrepreneurship education. Guided by the Competency Iceberg Model (Spencer and Spencer, 2008), the current study utilized LPA to explore latent subgroups within university teachers' IEE patterns. Additionally, multinomial logistic regression analysis was used to examine the factors associated with the different latent IEE groups.

The findings identified three distinct latent subgroups of IEE teachers, each characterized by uniform levels of competence across all eight IEEC dimensions: the High IEE-Competent Group (with consistently high scores on both unobservable psychological traits and observable instructional skills), the Average IEE-Competent Group (with balanced, above-average competence across all dimensions), and the Moderate IEE-Competent Group (with foundational levels of competence across all dimensions). Furthermore, two factors—entrepreneurship/business management experience and innovation-entrepreneurship research experience—were found to be significantly associated with teaching competency outcomes. Higher education IEE teachers with prior entrepreneurship/business management experience or IEE research experience were significantly more likely to be classified in the High IEE-Competent Group relative to teachers without such experience. These findings highlight that enhancing IEE competence among higher education teachers requires tailored interventions that focus on enriching their relevant professional experiences and nurturing key individual psychological characteristics.

Firstly, this study contributes to IEE scholarship by applying LPA to examine IEE competence among higher education teachers, identifying three distinct latent IEE competence subgroups: the High IEE-Competent Group, the Average IEE-Competent Group, and the Moderate IEE-Competent Group. Prior studies have applied approaches, such as back propagation neural network (Wang et al., 2023) and structural equation modeling (Yao, 2021) to evaluate the IEE outcomes and developmental pathways. However, these studies primarily model IEE variables based on observed variables and have not adequately accounted for the significant individual differences among educators (Liu et al., 2024). To address this gap, the present study developed the IEEC questionnaire to assess university IEE teachers' competencies via a rigorous process combining competency theory with in-depth participant interviews. Using this measure, LPA identified three latent subgroups of IEE competence across the eight theoretically derived dimensions: entrepreneurial mindset, professional mindset, ambition, optimism, self-efficacy, professional knowledge, enterprising skills, and teaching ability. This represents both a methodological innovation and valuable insights into the developmental pathways of IEE competencies, highlighting key areas for targeted faculty improvement programs. These findings can inform the design of more personalized and effective development initiatives, supporting the growth of university teachers' IEE competencies in ways that aligns with their unique needs and strengths.

Secondly, this study contributes to the empirical literature by verifying positive correlations between specific factors and teaching competencies, enabling more targeted improvements in IEE implementation. After identifying three distinct subgroups in IEE teacher competencies, we specifically examined how various experiences (such as teaching duration, entrepreneurship/business management experience, innovation-entrepreneurship research experience, and competition mentoring experience) correlate with each subgroup. The results showed that higher education IEE teachers with prior entrepreneurship/business management experience or IEE research experience were significantly more likely to be classified into higher-competence latent profiles relative to those without such experience. Notably, these associations align with international evidence that practical entrepreneurial experience and active scholarly research are core to developing authentic and effective IEE pedagogy (San-Martín et al., 2021). The study builds on existing frameworks that emphasize multi-level support systems, including institutional environments and individual entrepreneurial behaviors (Zhu and Shu, 2021; Zhu et al., 2023). Prior research has identified various instructional and personal factors influencing teacher competency (Liu et al., 2024), including cognitive-behavioral mediators (i.e., environmental education comprehension and teaching methods). Despite this, significant questions remain about how these elements collectively influence teaching outcomes. By identifying key factors of competence, the results provide evidence-based insights for improving the quality of IEE teaching. We recommend enriching university IEE teachers' entrepreneurship/business management experience and innovation-entrepreneurship research experience to enhance their IEE competencies.

## 5 Implications

The present study makes several noteworthy contributions to the field of IEE competency. These findings strengthen the foundation for advancing teaching competency research and clarifying the connection between instructional practices and personal experience. Firstly, we have expanded the competency iceberg model by incorporating a broader range of factors, including entrepreneurial mindset, professional mindset, ambition, optimism, self-efficacy, professional knowledge, enterprising skills, and teaching ability. From the theoretical perspective of IEE competency, this comprehensive approach offers a more nuanced understanding of the competencies required for effective teaching in IEE. It emphasizes not only the technical and professional skills but also the personal attributes and mindset crucial for fostering an entrepreneurial culture within the classroom. Secondly, this study developed the LPA framework for assessing teaching competency in IEE. By extending and deepening the application of LPA, we provided additional theoretical frameworks and subgroup classifications for university teachers in the context of IEE. Specifically, we identified three latent subgroups: a high IEE-competent group, an average IEE-competent group, and a moderate IEE-competent group. Thirdly, we identified key factors associated with teachers' competencies in IEE, including entrepreneurial experience and research involvement. University IEE teachers with prior experience in entrepreneurship management or innovation-entrepreneurship research were significantly more likely to be classified into higher instructional competencies in IEE compared to those without such experience. Overall, the use of an LPA approach deepens our theoretical understanding by identifying latent subgroups among university IEE teachers. The findings provide valuable IEE competency group classifications that can guide the development of more targeted teacher training and development strategies. In light of these insights, educational institutions should prioritize enhancing university IEE teachers' relevant practical experience to strengthen their teaching competencies in IEE.

## 6 Limitations

Several limitations of this study warrant discussion and suggest valuable directions for future research. First, the reliance on self-reported data for all measures introduces the potential for common method bias, and the cross-sectional research design precludes the ability to draw causal inferences about the relationships between variables. Additionally, the impact of teacher IEE competence on actual student entrepreneurial outcomes and learning experiences remains unexamined in the present study. Second, the study sample was predominantly recruited from Guangdong Province via the Wenjuanxing platform, which limits the generalizability of the findings to the broader population of Chinese higher education IEE teachers. Additionally, the substantial gender imbalance in the sample (65.15% female) was not statistically controlled for in the analyses and may represent a potential confounding variable. Finally, IEE teaching tenure was measured as a dichotomous variable rather than a continuous one, and the potential mediating and moderating roles of other variables such as institutional support, teacher training remain unexplored. Future research should employ continuous measures of key variables, adopt longitudinal research designs to examine causal relationships, and incorporate external institutional and contextual factors to provide a more comprehensive understanding of IEE competence development dynamics.

## 7 Conclusions

This study utilized a latent profile model to examine the latent subgroups of IEE competence among university teachers in China. The LPA results identified three distinct competency subgroups among IEE educators: high IEE-competent group, average IEE-competent group, and moderate IEE-competent group. This study further examined four categorical independent variables potentially associated with teaching competency: (1) teaching duration, (2) entrepreneurship/business management experience, (3) innovation and entrepreneurship research experience, and (4) competition mentoring experience. The analysis revealed that only two factors—entrepreneurship/business management experience and innovation-entrepreneurship research experience—were significantly associated with profile membership. The study underscores the pivotal role of university teachers in IEE and empirically validates specific factors correlate with their instructional effectiveness. These findings provide valuable insights for targeted faculty development programs aimed at enhancing teaching quality in this increasingly important educational field.

## Data Availability

The raw data supporting the conclusions of this article will be made available by the authors, without undue reservation.
