# Correction: The competence in innovation and entrepreneurship education among Chinese university teachers: a latent profile analysis

**DOI:** 10.3389/fpsyg.2026.1868080

**Published:** 2026-05-26

**Authors:** Wenyi Zhang, Huiqing Li, Xudan Zhang, Zhiying Yang, Yingzhi He

**Affiliations:** 1 School of Business Administration/Cantonese Merchant School/Innovation and Entrepreneurship School, Guangdong University of Finance and Economics, Guangzhou, China; 2 The Chinese University of Hong Kong, Shenzhen, Shenzhen, China; 3 School of Education, South China Normal University, Guangzhou, China

**Keywords:** higher education, innovation and entrepreneurship education, latent profile analysis, teacher competence, university teacher

Affiliation School of Business Administration/Cantonese Merchant School/Innovation and Entrepreneurship School, Guangdong University of Finance and Economics, Guangzhou, China was erroneously given as School of Business Administration/Cantonese Merchant School/Innovation and Entrepreneurship School, Guangdong, University of Finance and Economics, Guangzhou, China.

The **Acknowledgements** section was erroneously omitted from the published article. This section now reads: “We sincerely appreciate the reviewers and the editor for their thoughtful comments and suggestions. Their valuable insights have significantly enhanced the quality of this work. Furthermore, we would like to express our sincere gratitude to Qing Zeng, a Ph.D. student at Beijing Normal University, for her valuable assistance with the statistical analyses.”

The original version of this article has been updated.

